# The Correctness of the Simplified Bernoulli Trial (SBT) Collision Scheme of Calculations of Two-Dimensional Flows

**DOI:** 10.3390/mi12020127

**Published:** 2021-01-26

**Authors:** Kiril Shterev

**Affiliations:** Institute of Mechanics, Bulgarian Academy of Sciences, Acad. G. Bontchev St., bl. 4, 1113 Sofia, Bulgaria; kshterev@imbm.bas.bg; Tel.: +359-2-9792007

**Keywords:** DSMC, SBT, two-dimensional flows, pressure-driven, shuffling PPC, minimal and maximal PPC, gas-mixing

## Abstract

Micro-electromechanical systems (MEMS) have developed rapidly in recent years in various technical fields that have increased their interest in the Direct Simulation Monte Carlo (DSMC) method. In this paper, we present a simple representation of the DSMC collision scheme and investigate the correctness of the Simplified Bernoulli Trial (SBT) collision scheme for the calculation of two-dimensional flows. The first part of the collision scheme, which determines collision pairs, is presented following the derivation of the expression for the mean free path and using the cumulative distribution function. Approaches and conclusions based on one-dimensional flows are not always directly applicable to two- and three-dimensional flows. We investigated SBT correctness by using the two-dimensional pressure-driven gas flow of monoatomic gas as a test case. We studied the influence of shuffling of the list of particles per cell (PPC) before the collision scheme’s execution, as well as the minimal and maximal number of PPC, on the correctness of the solution. The investigation showed that shuffling and the number of PPC played an important role in the correctness of SBT. Our recommendations are straightforwardly applicable to three-dimensional flows. Finally, we considered the mixing of two gases and compared the results available in the literature.

## 1. Introduction

The Direct Simulation Monte Carlo (DSMC) approach is a basic numerical method employed for the study of rarefied gas flows by using a discrete set of a finite number of simulators (particles) modeling the behavior of the molecules of a real gas. Bird proposed DSMC in the late 1960s (see [1]). The application of DSMC takes place between molecular dynamics and the continuum approach to describe fluid. Molecular dynamics are applicable to the modeling of a very small number of gas molecules. As the number of molecules increases, the computational resources required increase many times, and it is impossible to calculate problems in the region of even 1 micrometer at a standard pressure and temperature. On the other hand, classical continuum approaches based on Navier–Stokes equations are limited in terms of reducing the size of the devices due to the predominant influence of micro-phenomena that cannot be described by employing a continuum approach. One has to use numerical methods that solve the Boltzmann equation, such as the Discrete Velocity Method [2,3]. The DSMC is the most commonly used approach and naturally includes particles’ impulses on solid surfaces in the simulation process.

A collision scheme with an accuracy independent of the number of particles per cell (PPC) is important for many applications. The mesh around moving geometries and deformable surfaces has to adapt continuously. An example of such a problem is Fluid–Structure Interaction (FSI). FSI is key to resolving many physical problems that cannot be handled separately from a structural or fluid point of view (see [4]). Shterev and Manoach [5], used for the first time, according to the authors’ knowledge, molecular approach (DSMC) to model rarefied gas in the FSI algorithm. They used Stefanov’s Simplified Bernoulli-Trial (SBT) collision scheme [6,7].

The basic ideas underlying the Direct Simulation Monte Carlo (DSMC) method and collision schemes have been examined in the papers of Roohi and Stefanov [8] and Stefanov [9].

The most popular collision scheme is Bird’s No Time Counter (NTC) [1]. This approach requires more than 10 (often 20–30) PPC to obtain correct results. Satisfying the minimal requirements of the number of PPC of NTC prevents repeated collisions as a source of error. NTC computational requirements are proportional to $N^{\left(l\right)}$, where $N^{\left(l\right)}$ is the number of PPC and *l* is the index of a mesh cell. Modern NTC variants, such as the nearest neighbor (NN) method have been widely analyzed and discussed [10,11,12,13]. Another famous collision scheme of this group is the Majorant Frequency scheme (MFS) developed by Ivanov and Rogasinsky [14]. The advantage of collision schemes of this group is that the computational requirements are proportional to $N^{\left(l\right)}$, while their disadvantage is the deviation of the solutions that occurs when the minimal requirement of PPC is violated.

The requirement of a minimal number of PPC leads to continuously monitoring the number of PPC and precise remeshing when the number of PPC is not sufficient. Often, remeshing demands significant calculations that make the whole simulation slower. This can be avoided if the collision scheme obtains correct results for an arbitrary number of PPC.

Bernoulli Trial-based collision models obtain correct results with a small number of PPC. Collision algorithms based on the Bernoulli Trial (BT) scheme were proposed by Belotserkovskii and Yanitskiy [15] and Yanitskiy [16]. They avoided repeated collisions that eliminate this source of error. In accordance with the Kac stochastic equation [17], BT checks each particle pair. The number of checks is $N^{\left(l\right)}(N^{\left(l\right)}-1)/2$. BT suffers from high computational costs of a quadratic order, according to $N^{\left(l\right)}$. Stefanov proposed the Simplified Bernoulli Trial (SBT) collision scheme [6,7]. SBT reduces the number of operations of the BT collision scheme. The SBT computational requirements are proportional to $N^{\left(l\right)}$, which is of the same order of computational requirements of NCT. That has increased the interest in BT-based schemes. In papers [18,19] the authors investigated SBT’s accuracy over a broad range of discretization parameters and devoted efforts to reducing the computational resources of BT-based collision models. Taheri et al. [18] studied the accuracy of SBT and NTC collision schemes in one-dimensional (1D) Fourier and Couette problems. The SBT convergence behavior for the heat flux was investigated over a broad range of discretization parameters. SBT showed an excellent accuracy, even with 0.5 PPC, while NTC could not obtain correct results with 5 PPC. The authors reported that the SBT scheme works fine if the exchange of particles between neighboring cells occurs quite often. They pointed out that the effective parameter in the convergence is the cell size to time step ratio, ∆*x*/∆*t* (∆*x = dx/λ* and ∆*t = dt/t_c_*, where *λ* is the gas mean free path and *t_c_* is the mean collision time), which should be properly adjusted for any specific test case. Roohi et al. [19] have devoted efforts to reducing the computational requirements of the SBT collision scheme. They presented a generalized form of the Bernoulli Trial collision scheme (GBT). The GBT technique reduces the number of selected pairs for a possible binary collision while ensuring a correct collision frequency using a modified collision probability formula. GBT was shown to further improve the computational efficiency of the BT-based collision models compared to the standard NTC and NN collision models. The authors also pointed out the importance that the ratio ∆*x*/∆*t* is regulated, being smaller than 2 in all simulations.

SBT guarantees correct results for a small number of PPC, while NTC requires more than 10 (often 20–30) PPC. In the considered papers, SBT and its correctness are studied in detail for 1D flows, while in the present study, we investigated the SBT correctness of two-dimensional (2D) flows. We selected the SBT scheme for our study as a representative of BT-based schemes.

The SBT-CDF collision scheme using the cumulative distribution function (CDF) and following the survival equation is presented in this paper. The aim is to present the DSMC collision scheme to the already wider audience of non-professionals in this field in a simpler and accessible way. We investigated the SBT collision scheme correctness for multidimensional flows. There is a fundamental difference between 1D and 2D cases from a numerical methods point of view. Successful approaches and conclusions for 1D problems are not always applicable to 2D and 3D problems. This motivated us to investigate the SBT collision scheme correctness for 2D flows. The presented considerations for 2D flows can be straightforwardly extended to 3D flows. 2D pressure-driven flow in a long microchannel was used as a test case because it is simple and axisymmetric, and the pressure varies significantly. We studied the influence of shuffling the list of PPC and the number of PPC on the solution correctness in detail. Finally, we considered binary gas mixing and the obtained results were compared to those available in the literature.

## 2. DSMC Collision Scheme with Explanations Based on the Cumulative Distribution Function

In recent years, the rapid development of micro-electromechanical systems (MEMS) has increased the interest in DSMC as a dominant method for modeling the gas in a transition regime. DSMC has been used and developed by professionals in this field. This has left a mark on the literature. In general, DSMC literature contains specific information that forces interested non-professionals to search and read many additional books. To be clear, with regard to presenting the basic concept of the collision scheme to a wider audience, the explanation begins with preliminary explanations. It is recommended that the reader is familiar with the Cumulative Distribution Function (CDF, see [20], Chapters 1.2.2 and 2.1.2) and the derivation of the survival equation (see [21], Chapter 10-3 Collision cross section. Mean free path). The survival equation has been used to derive the expression for the mean free path of the gases. DSMC simulates gases by maintaining the given velocity distribution and collision frequency (mean free path).

The DSMC method separates the motion of gas molecules into ballistic and collision parts. The DSMC collision scheme without chemical reactions consists of two steps. The first step determines particles that collide. The second step determines post-collision velocities. In this paper, we consider the first step of the collision scheme that determines collision pairs. The presented explanation of the collision scheme uses the definition for the mean free path and the bullet target idea that has been used in the derivation of the equation of the mean free path.

### 2.1. Preliminary Considerations

**Figure 1:**
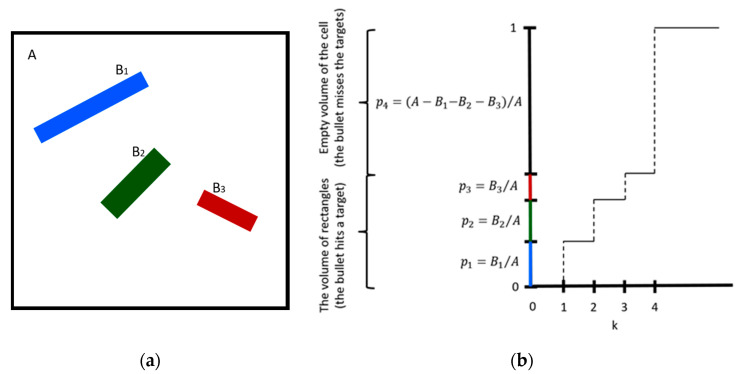
“Bullet-target” examples with the three targets B1, B2, and B3 in a square A (**a**) and the corresponding Cumulative Distribution Function (CDF) (**b**).

The Monte Carlo method has an important advantage over deterministic methods. To illustrate this, we considered the following “bullet-target” example. The targets are three rectangles placed in a cell (see Figure 1a). The area of the cell is equal to *A*, while the areas of the rectangles are equal to *B*_1_, *B*_2_, and *B*_3_. The areas with indices *k* = 1, 2, 3, and 4 correspond to the area of *B*_1_, *B*_2_, *B*_3_, and (*A − (B*_1_ *+ B*_2_ *+ B*_3_)), respectively. We illustrated a simulation of shooting at a square *A* with targets *B_1_*, *B_2_*, and *B_3_* using the Monte Carlo method. The distribution of hits at the square *A* is uniform. Therefore, the probabilities of hitting targets *B*_1_, *B*_2_, *B*_3_, and the area of the empty space in *A* are proportional to the area, being *p*_1_ = *B*_1_/*A*, *p_2_* = *B*_2_/*A*, *p*_3_ = *B*_3_/*A*, and *p_4_* = (*A − (B*_1_ *+ B*_2_ *+ B*_3_))/*A*, respectively. To determine which target a bullet hits, we used the multiple-outcome cumulative distribution function (CDF). Let *R* be a random variable. The CDF of *R* was defined as CDF (*k*) = Pr(*R* ≤ *k*). Figure 1b shows the CDF of *k*. Inverting the CDF determines *k*. Let U ∼ U (0, 1) be a uniform (0, 1) sample. Starting from the point (0, U) (on the *y*-axis), proceed to the right until you encounter a jump in the CDF, represented by a vertical dashed line segment. Now proceed down to the *x*-axis, and return this value as the selection. As indicated along the *y*-axis, “1” is selected with *p*_1_ probability, since its interval occupies this fraction of the unit interval. Similarly, “2” is selected with *p*_2_ probability, since its interval occupies this fraction of the unit interval, and so on. Using CDF, we simulated shooting at the target *A* and which area the bullet hits was returned as the result. The Monte Carlo simulation generated one uniformly distributed random number and one calculation of the CDF per shoot. An important part was that events were not equally likely. The probability of hitting a rectangle depends on the rectangle’s area. Target places and exact shapes were not considered. The number of calculations depends on the number of areas. On the other hand, a geometrical approach has to determine which area was hit, taking into account shapes and places of the areas. The number of calculations increases with the complexity of the shapes of the areas, their number, and their dimension. The Monte Carlo simulation for the 3D case would be similar to the 2D case presented, substituting areas with volumes. After all, Monte Carlo methods “substitute” the problem with generating a random number and simple calculations, while geometrical methods need to maintain all information on the considered problem. Monte Carlo’s advantage is essential when the number of “targets” and the complexity of their shapes increase.

The relation of the presented problem to the DSMC collision scheme is straightforward, and is considered in the next section.

### 2.2. DSMC Collision Scheme with Explanations Based on the Cumulative Distribution Function

In this paper, we present the first step of the collision scheme that determines collision pairs. The collision schemes only consider nearly placed particles. The most widely used approach is separating the computational domain with a mesh and considering collisions between the particles in a cell of a mesh. In the collision procedure, the particle’s coordinates are not considered. Therefore, the particles from other cells are not taken into account. The time step has to be sufficiently small to guarantee that none of the particles will fly over any cell without execution of the collision procedure. This is the so-called Courant–Friedrichs–Lewy (CFL) condition [22].

The idea of the presented explanation of the collision scheme arises from the derivation of the mean free path of hard sphere gas. In DSMC, one simulator particle (for short, particle) represents many real molecules or atoms. This assumption significantly reduces the computational resources required. DSMC fills the gap between molecular dynamics and continuum models, where molecular dynamics requires impossibly large amounts of computational resources and continuum models are no longer valid.

**Figure 2:**
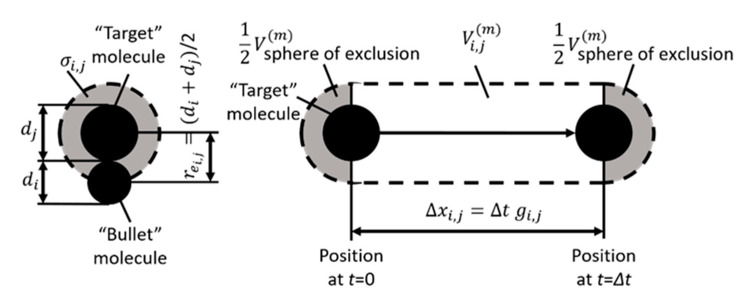
Sphere of exclusion, collision cross section (σ), and collisional cylinder ($V_{i,j}^{\left(m\right)}$) of two molecules with different diameters: *d_i_* (“bullet” molecule) and *d_j_* (“target” molecule).

The collision scheme uses the list of particles in the considered cell and the cell’s volume. Let us refer to one of the simulator particles as the “bullet” particle and to the others as “target” particles. A collision occurs whenever the distance between the centers of the molecules becomes equal to $r_{e_{i,j}}$. If the molecules *i* and *j* are considered as hard spheres of diameter *d*_i_ and *d*_j_, respectively, the minimum distance between the centers of two molecules, as shown in Figure 2, is equal to $r_{e_{i,j}}=(d_{i}+d_{j})/2$. In effect, the center of each molecule is excluded by the other from a sphere of radius *r_e_*, known as the “sphere of exclusion.” Since it is only the center-to-center distance that determines a collision, it does not matter whether the target is large and the bullet small, or vice versa. We may therefore consider the bullet molecule to shrink to a point at its center, and the target molecule to occupy the entire sphere of exclusion, of radius *r_e_*.

**Figure 3:**
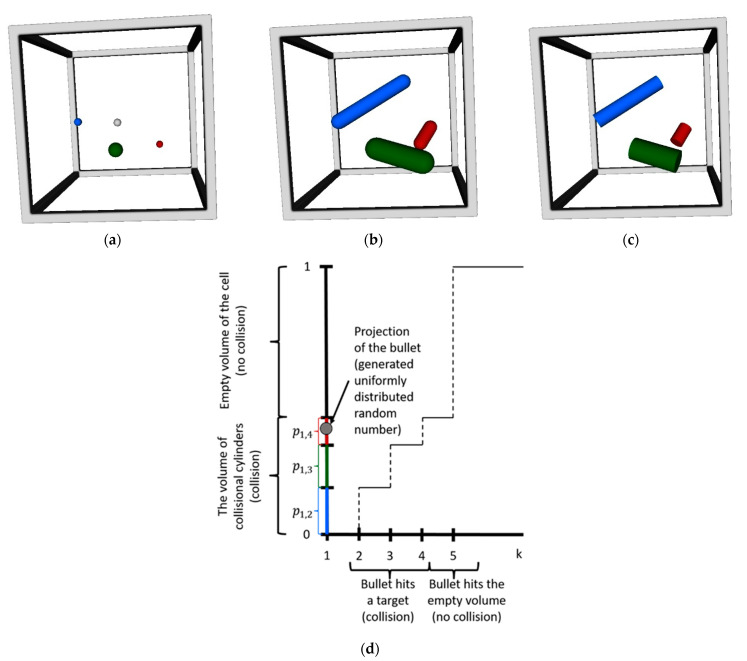
Example of the collision procedure of particles in the cell. (**a**) shows the particles in a cell; (**b**) shows the volumes occupied by target molecules with relative velocities to the bullet molecule; (**c**) shows the volumes through which each of the targets flies relative to the bullet, representing the so-called “collisional cylinders”; and (**d**) shows the CDF for considered molecules.

Now consider the molecules in a cell (see Figure 3). All molecules have different diameters. We choose the gray molecule to be bullet molecule and others (blue, green, and red) to be target molecules. The target molecules are moving with relative velocities to the billet for a period of time Δ*t* (time step). The probability that the bullet hits at least one of the targets is the ratio of the volumes through which the targets fly relative to the bullet to the volume of the cell. The derivation of the probability is as follows. The volume that occupies each of the targets flying relative to the bullet for a considered time is:

(1) $${V_{all}}_{i,j}^{\left(m\right)}=g_{\;i,j}\sigma_{\;i,j}\Delta t+V_{\mathrm{sphere}\;\mathrm{of}\;\mathrm{exclusion}}^{\left(m\right)},\;\;\;\;\;\;\mathrm{for}\;\;i=0,1,2,\;\;\;\mathrm{and}\;\;\;j=i,i+1,3,$$

where $\sigma_{i,j}=\pi {r_{e}}_{i,j}^{2}$ is the collision cross section of the bullet molecule *i* and target molecule *j*; $g_{\;i,j}=\sqrt{{\left(u_{i}-u_{j}\right)}^{2}+{\left(v_{i}-v_{j}\right)}^{2}+{\left(w_{i}-w_{j}\right)}^{2}}$ is the relative velocity of a molecule pair (*i, j*); *u*, *v*, and *w* are the velocities of the molecule along the *x*-, *y*-, and *z*-axis, respectively; and $V_{\mathrm{sphere}\;\mathrm{of}\;\mathrm{exclusion}}^{\left(m\right)}=\frac{4}{3}\pi {r_{e}}_{i,j}^{3}$ is the volume of the sphere of exclusion. These volumes are shown in Figure 2 (the right part) and Figure 3b. If we maintain the volume of the sphere of exclusion of the targets when Δt→0, the collision probability approaches a constant greater than zero. From a physical point of view, this means that the particles can collide each other when they are not moving. As this is physically unrealistic, the volume of a sphere of exclusion has to be removed from the equation. For Δt→0, the collision probability approaches zero (the particles do not collide). Therefore, the volume through which each of the targets fly relative to the bullet for a considered time is the so-called “collision cylinder” of a molecule pair (*i*, *j*):

(2) $$V_{i,j}^{\left(m\right)}=g_{\;i,j}\sigma_{i,j}\Delta t,\;\;\;\;\;\;\mathrm{for}\;\;i=0,1,2,\;\;\;\mathrm{and}\;\;\;j=i,i+1,3.$$

It should be added that condition $V_{i,j}^{\left(m\right)}/V^{\left(l\right)}\leq 1$ is also physically reasonable for the molecules of a rarefied gas [9], where $V^{\left(l\right)}$ is the volume of the considered cell *l*.

Figure 3c shows the volumes of collisional cylinders. One can derive the CDF function for this case as was done in the previous section (Figure 1) and to obtain the equivalent Monte Carlo problem (see Figure 3d). Volumes of “collisional cylinders” presented in Equation (2) are mapped to one-dimensional space by normalization to a volume of a cell ($V^{\left(l\right)}$). In this way, we can obtain the probability that the bullet *i* collides with target *j*:

(3) $$p_{i,j}^{\left(m\right)}=V_{i,j}^{\left(m\right)}/V^{\left(l\right)}.$$

One can see that the collision probabilities depend on pairs of molecules and are not equally likely. In the collision step, we do not consider the actual place of the molecules. Therefore, we do not consider the case that a particle can leave the cell for a considered time step, even when the particle is close to the cell’s edge and will leave it at the next execution of the moving procedure. The Courant–Friedrichs–Lewy (CFL) condition and $V_{i,j}^{\left(m\right)}/V^{\left(l\right)}\leq 1$ guarantee that the sum of the volumes of the collisional cylinders is sufficiently small compared to the cell size and the sum of collision probabilities will be less than 1. In the collision procedure, the coordinates of the particles are not considered; therefore, we can place a bullet molecule at random coordinates in a cell volume. The coordinates of the bullet can be considered as constant for this time step as the velocities of the particles are constant, and target molecules are moving relative to the fixed bullet molecule. The projection of the bullet molecule is obtained by generating one uniformly distributed random number (see Figure 3d). In the considered case, the bullet molecule will hit the target molecule 3. Here, it should be point out that by using the presented approach with one random number, we can determine everything for the considered event, depending on the projection of the bullet:

1. If the bullet is projected into an empty volume, there will be no collision, and
2. If the bullet hits any one of the targets, then
  2.1. The hit target and bullet are collision pairs, and
  2.2. We can determine when the collision occurs (considering the ratio of the travelled path to the collision to the path of the time step).

Each DSMC simulator particle represents many real molecules. The volume of collisional cylinders ($V_{i,j}^{\left(m\right)}$) in terms of the probability has to be multiplied by a number of real molecules that the simulated particle represents (the weight of simulated particles, ω). We obtained the following equations for the volumes of collisional cylinders and probability for DSMC:

(4) $$V_{i,j}=\omega g_{\;i,j}\sigma_{i,j}\Delta t,\;\;\;\;\;\;\mathrm{for}\;\;i=0,1,2,\;\;\;\mathrm{and}\;\;\;j=i,i+1,3,$$

(5) $$p_{i,j}=V_{i,j}/V^{\left(l\right)}.$$

We defined the multiple-outcome CDF using probability (5) as

(6) cdf(j)=Pr(R≤j),

where *R* is a random variable and $\sum p_{i,j}$ must satisfy the condition

(7) $$Prob\{\sum p_{i,j}\geq 1\}\to 0.$$

The presented derivation of the collision scheme uses the CDF function to calculate the probability and follows the SBT collision scheme procedure. For brevity in the paper, it was named SBT-CDF. The correctness of SBT for various numerical parameters was investigated. The results show that SBT and SBT-CDF obtained the same solution (see Section 3.1.5 of the paper). SBT-CDF requires a greater amount of calculation compared to SBT, but the difference in the computational time was not significant. The calculation times are presented in Section 3.1.4 and discussed in the discussion section.

**SBT collision procedure applied to cell (*l*)**

Shuffle the PPC list.

A sequence of pairs $i=1,\ldots ,(N^{\left(l\right)}-1)$ is chosen from $N^{\left(l\right)}$ particles in cell *l* as follows:

- The first particle *i* is the particle with index *i* in the particle list created for cell *l*, and
- The second particle $j\in \left[i+1,N^{\left(l\right)}\right]$ is chosen with probability $p_{1}=1/k$ from $k=(N^{\left(l\right)}-i)$ particles on the list after particle *i*.

Particle pair (*i*,*j*) is checked for collision with the probability

(8) $${\hat{W}}_{i,j}=k\frac{\omega g_{i,j}\sigma_{i,j}\Delta t}{V^{\left(l\right)}},$$

where ${\hat{W}}_{i,j}$ must satisfy the condition

(9) $$Prob\{{\hat{W}}_{i,j}\geq 1\}\to 0.$$

If the collision is accepted, then the velocities $\left(c_{i},c_{j}\right)$ are changed to the post-collision values $\left(c\prime_{i}c\prime_{j}\right)$; otherwise, they remain unchanged.

**SBT-CDF collision procedure applied to cell (*l*)**

Shuffle the PPC list.

A sequence of pairs $i=1,\ldots ,(N^{\left(l\right)}-1)$ is chosen from $N^{\left(l\right)}$ particles in cell *l* as follows:

- The first particle *i* is the particle with index *i* in the particle list created for cell *l*, and
- Calculate *j* using CDF (6), taking into account the particles with indexes $j\in \left[i+1,N^{\left(l\right)}\right]$.

If the collision is accepted, the velocities $\left(c_{i},c_{j}\right)$ are changed to the post-collision values $\left(c\prime_{i},c\prime_{j}\right)$; otherwise, the bullet hit the empty space and the collision does not happen.

The shuffling of the list of PPC was included in SBT and SBT-CDF. Particle rearrangement in the collision procedure has been mentioned in [19], but without results showing its influence on the solution.

## 3. Results

We investigated SBT and SBT-CDF correctness using pressure-driven flow as a test case. According to the results, we proposed recommendations. The other presented problem is gas mixing.

### 3.1. Pressure-Driven Gas Flow in a Microchannel

The pressure-driven gas flow in a microchannel was used as a test case. Pressure-driven flow is a relatively simple flow that has an analytical solution for the continuum slip regime, pressure varies significantly, and it is axisymmetric. We used these properties to investigate the correctness of SBT and SBT-CDF collision schemes.

**Figure 4:**
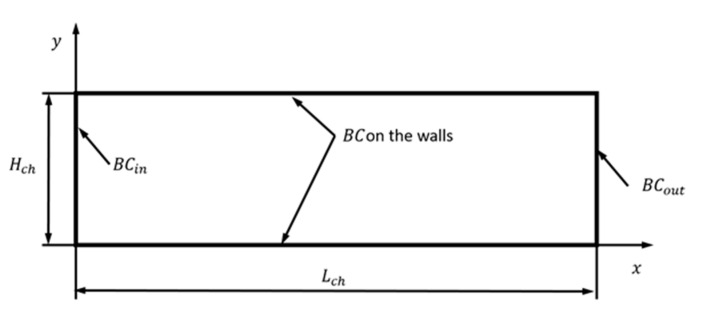
Geometry of a microchannel with length *L_ch_* and height *H_ch_*.

Figure 4 shows the flow geometry of a microchannel. The ratio of the pressure at the inlet to the outlet is $P=p_{in}/p_{out}=3$ and the Knudsen number is *Kn* = 0.05 ($Kn=\lambda /H_{ch}$, where λ is the mean free path of the gas molecules at the reference pressure and the reference temperature and *H_ch_* is the channel height). The aspect ratio of the channel is $A=L_{ch}/H_{ch}=50$. The reference pressure is the pressure at the channel outlet (*p_out_*), the reference temperature is the temperature at the channel inlet (*T_in_*), the temperature of the channel walls is equal to *T_in_*, and the reference velocity is the thermal velocity of gas molecules ($V_{th}=\sqrt{2RT_{in}}$, where *R* is the mass-specific gas constant).

The inlet boundary conditions BC_in_ at *x* = 0 are

(10) $$p=p_{in},\;\frac{\partial u}{\partial x}=0,\frac{\partial v}{\partial x}=0,\;T=T_{in}.$$

At the outlet *x* = *L_ch_*, we impose the following boundary conditions BC_out_:

(11) $$p=p_{out},\;\frac{\partial u}{\partial x}=0,\frac{\partial v}{\partial x}=0,\;\frac{\partial T}{\partial x}=0.$$

The diffuse reflection boundary conditions were used at the microchannel walls. The DSMC method used a uniform mesh with 2000 × 40 cells and 5.7 × 10^6^ particles. In our considerations, we used the DSMC method with Transient Adaptive Sub-cell (TAS) [23] refinement. The TAS refinement used here follows the steps presented in [24]. The TAS technique consists of two elements. First, the cells of the basic grid were divided into sub-cells using a local uniform Cartesian mesh. The condition for dividing cells into sub-cells was determined by the requirement of the minimal average number of particles per sub-cell within a time step, which reads as follows:

(12) $${level}_{I,J}=\left\lfloor \sqrt{PPC_{I,J}^{\left(basic\right)}/PPC_{\min}}\right\rfloor ,$$

where $level_{I,J}$ is a TAS level of divisions, representing an integer number that denotes the number of sub-cells in each of the directions of cell *I*,*J*, i.e., for the 2D case, the local mesh is $level_{I,J}\times level_{I,J}$ cells, $PPC_{I,J}^{\left(basic\right)}$ is the number of particles per basic cell *I*, *J*, and $PPC_{\min}$ is the minimal average number of particles per sub-cell. This criteria keeps the number of particles per cell as close to constant as is possible.

Second, the maximum *level* ($level_{\max}$) was set at the beginning of calculations and the time step was adjusted to the minimal sub-cell size. The *level* array was evaluated once every $level_{\max}$ time step. The value of the next time step was determined to be in agreement with the Courant–Friedrichs–Lewy (CFL) condition, i.e., the time step in each sub-cell was determined to be equal in terms of the mean to the time of particle flight across the sub-cell. Therefore, for a sub-cell system with different levels of divisions, the global time step was the smallest value △t calculated from the CFL condition.

Firstly, we present the results related to the code correctness: The shuffling of the PPC list step in the collision scheme; the minimal number of PPC in 2D flows; and the influence of a constant number of PPC on the solution. After that, we present code validation of the considered case.

#### 3.1.1. Influence of the Shuffling of the PPC List in the Collision Scheme on the Solution

The shuffling of the lists of PPC is an important step in the collision schemes used. Particle rearrangement in the collision procedure was mentioned in [19], but without results showing the influence on the solution. The shuffle of the list of PPC can be applied once before the collision procedure or at every change of the first particle (*i*). We did not find a difference between these approaches in the considered problem. Nevertheless, the difference between the collision procedure with and without shuffling the list of particles was notable. The results were obtained with an algorithm that sorts PPC lists into groups by the cell from which particles fly from. For the 2D case, the groups of particles in the cell (*I*, *J*), where *I* is the index of the cell along the *x*-axis and *J* is the index of the cell along the *y*-axis, are formed as follows:

1st group of particles were in cell (*I* − 1, *J* − 1);
2nd group of particles were in cell (*I*, *J* − 1);
3rd group of particles were in cell (*I* + 1, *J* − 1);
4th group of particles were in cell (*I* − 1, *J*);
5th group of particles were in cell (*I*, *J*);
6th group of particles were in cell (*I* + 1, *J*);
7th group of particles were in cell (*I* − 1, *J* + 1);
8th group of particles were in cell (*I*, *J* + 1);
9th group of particles were in cell (*I* + 1, *J* + 1).

In this way, the algorithm sorts the particles at every time step.

**Figure 5:**
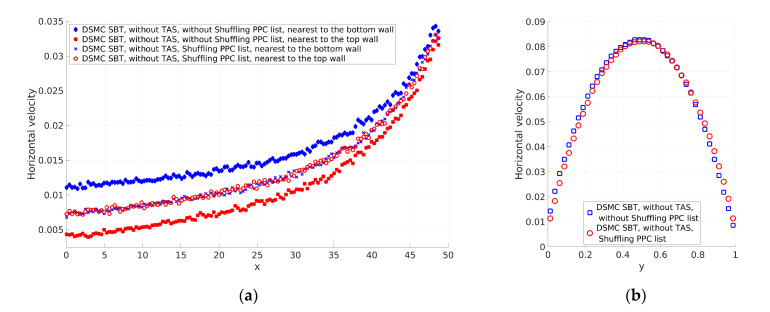
Horizontal velocity (u) of the gas obtained without Transient Adaptive Sub-cell (TAS) and without and with shuffling of the particles per cell (PPC) list: (**a**) Along the channel nearest to the bottom and top channel walls and (**b**) normal to the channel axis at the middle of the channel (*x* = *L_ch_*/2).

**Figure 6:**
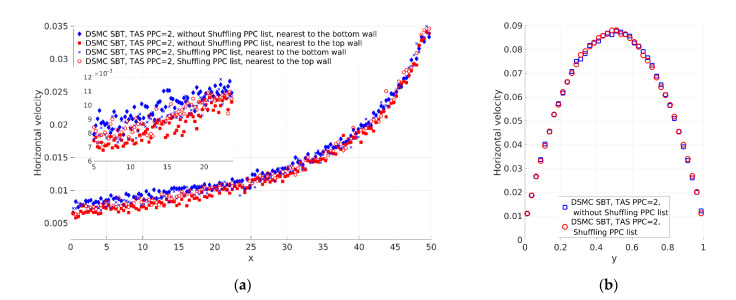
Horizontal velocity (u) of the gas obtained with TAS with PPC = 2 and without and with shuffling of the PPC list: (**a**) Along the channel nearest to the bottom and top channel walls and (**b**) normal to the channel axis at the middle of the channel (*x* = *L_ch_*/2).

Two cases with significant differences in the number of PPC were considered. The other important parameter was the time step. The time steps for the considered cases were different and satisfied the Courant–Friedrichs–Lewy (CFL) condition [22], i.e., the time step should be sufficiently small to guarantee that none of the particles can fly over a distance larger than the cell size without execution of the collision procedure. Figure 5a and Figure 6b show the horizontal velocity of the gas nearest to the top and bottom channel walls. The first case is that not using TAS (Figure 5). The number of PPC varies from approximately 100 at the channel inlet to approximately 34 at the channel outlet. The differences between the horizontal velocities at the channel walls are significant at the channel inlet and decrease at the channel outlet. The numbers of PPC at the channel inlet are larger than the numbers of PPC at the channel outlet. Additionally, the velocities at the channel inlet are lower than the velocities at the channel outlet. Finally, the time step was the same for the computational domain and the CFL condition was satisfied. As a result, the larger number of PPC and slower velocities lead to smaller changes in the lists at the channel inlet compared to the changes in the lists at the outlet. This was the reason why we obtained larger deviation at the channel inlet compared to the channel outlet. The profiles of velocities at the channel walls have to be the same as the flow is symmetrical. When including shuffling of the lists as a preliminary step, we obtained the same velocity profiles at the channel walls along the channel (Figure 5a), and the velocity profile normal to the channel was symmetric (see Figure 5b). The second case used TAS. The considerations were confirmed when we used TAS with the number of PPC equal to 2 (see Figure 6). TAS kept the number of PPC smaller than the case without TAS, PPC changed more intensively, and SBT obtained results with a lower deviation of the velocity field. The obtained solution using the shuffling of PPC lists also demonstrated expected axis symmetry (Figure 6b). As the velocity profiles in Figure 6 are close, to compare them, we calculated the difference between the mean horizontal velocity along the channel of cells nearest to the channel walls. The difference without shuffling of the list of particles is $\left|{\bar{u}}_{top}^{}-{\bar{u}}_{bottom}^{}\right|=1.17\times 10^{-3}$, while the difference with shuffling is $\left|{\bar{u}}_{top}^{\left(Sh\right)}-{\bar{u}}_{bottom}^{\left(Sh\right)}\right|=6.76\times 10^{-5}$. The shuffling improved the axis symmetricity of the flow by nearly two orders of magnitude. After all, using the shuffling of PPC lists as a preliminary step produced symmetric results of the SBT collision scheme with a different number of PPC.

The shuffling of the PPC list improves the DSMC solution. We tested other preliminary manipulations of the PPC list: Ascending sorting of the PPC list according to the probability of the collision scheme; descending sorting of the PPC list according to the probability of the collision scheme; and ascending sorting of the PPC list and shuffling (as a result, the PPC list was shuffled). The preliminary step with ascending and descending sorting of the PPC list obtained symmetrical profiles of the horizontal velocity, but the temperature of the gas in the channel was higher. The case of ascending sorting of the PPC list and shuffling obtained the same results as shuffling of the PPC list without sorting. As shuffling without sorting requires less computational resources, we recommend it.

Figure 5b and Figure 6b show the difference in the horizontal velocities at the channel center line (*y* = *H_ch_*/2). This difference is studied in Section 3.1.3.

The SBT and SBT-CDF collision schemes obtained the same results and the considerations regarding shuffling of the PPC lists for SBT are completely relevant to SBT-CDF.

#### 3.1.2. The Minimal Number of PPC in 2D Fluid Flows

**Figure 7:**
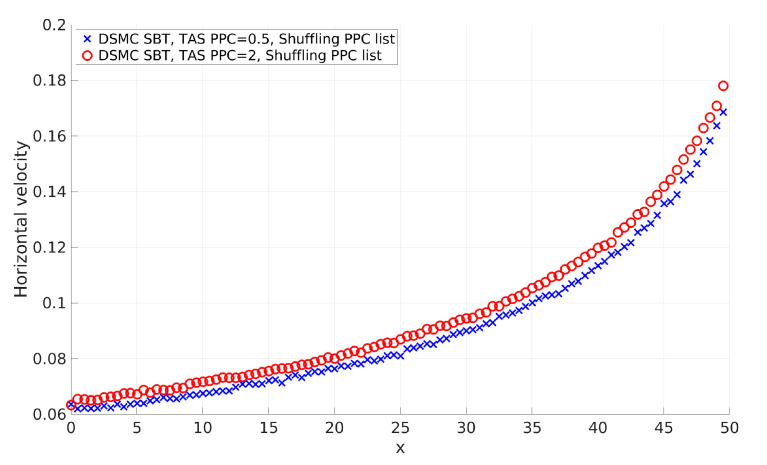
Horizontal velocity (u) of the gas of pressure-driven flow obtained with TAS with PPC = 0.5 and TAS with PPC = 2.

Figure 7 shows a comparison of profiles for 2D pressure-driven flow with PPC = 2 and PPC = 0.5. The case with PPC = 2 obtained the correct result (see validation in Figure 10), while the result of PPC = 0.5 deviated and corresponds to the solution with lower collision frequencies (greater mean free path). Similar deviation of 2D cavity flow results were presented in [7].

#### 3.1.3. Influence of the Number of PPC on a Solution

**Figure 8:**
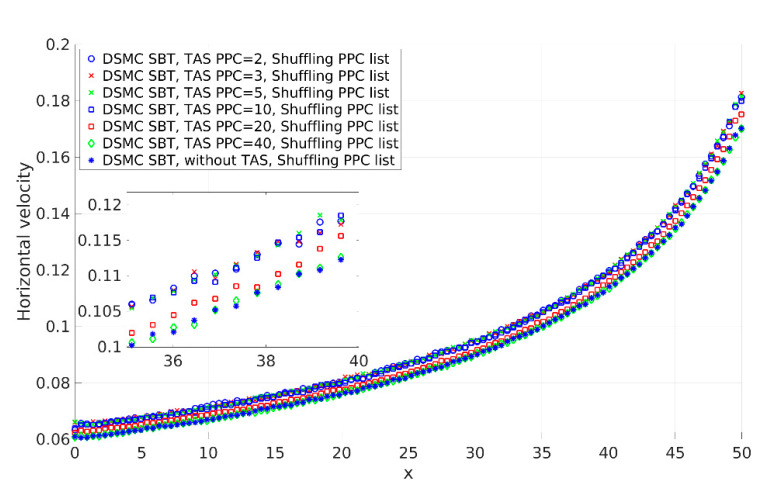
Horizontal velocity (u) of the gas of pressure-driven flow obtained with the Simplified Bernoulli Trial (SBT) and TAS with PPC values equal to 2, 3, 5, 10, 20, and 40 and without TAS.

We investigated the influence of the number of PPC on the correctness of the solution. Pressure-driven flow is relatively simple flow and the pressure varies significantly. Different pressures with equal temperatures in DSMC correspond to different numbers of particles per volume. The results show correspondence between the correctness of the solution and keeping the average number of PPC close to constant. Figure 8 shows profiles of the horizontal velocity (u) of the gas obtained with SBT and TAS with PPC = 2, 3, 5, 10, 20, and 40, and without TAS. The profiles obtained using TAS and PPC from 2 to 10 coincide and they are correct. Here, again for validation, we used SBT with TAS and PPC = 2 as its profile agrees with the NTC collision scheme (see Figure 10). When PPC increases to over 10, the velocity profiles deviate from the correct solution and tend to the profile obtained without TAS.

**Figure 9:**
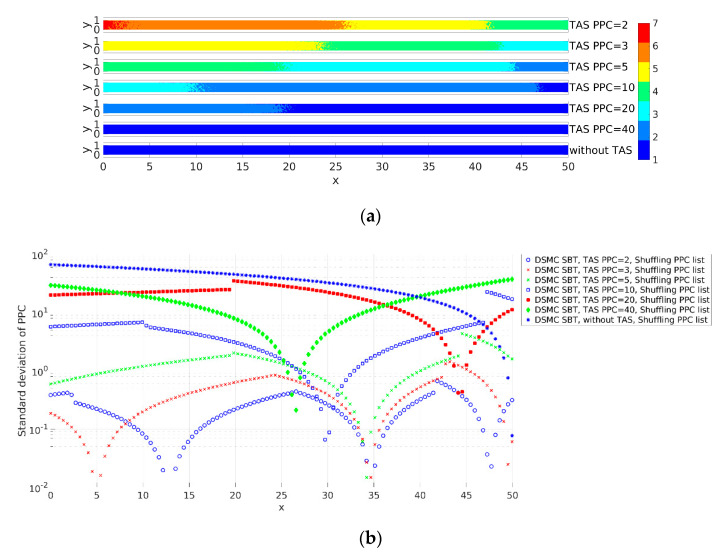
The smoothness of the adaptive mesh for considered cases of PPC equal to 2, 3, 5, 10, 20, and 40 and without TAS, where (**a**) shows the TAS level and (**b**) shows the standard deviations of PPC included in the collusion procedure along the channel.

Figure 9 shows the smoothness of the adaptive mesh that helped us to illustrate the number of particles included in the collision procedure. TAS is applied at every cell of a basic mesh and after TAS, each of the obtained sub-cells contains particles that are included in the collision procedure in this cell. Figure 9a shows the TAS levels in the computational domain. Figure 9b shows the deviation of the number of PPC included in the collision procedure.

We evaluated the smoothness of the adaptive mesh using standard deviation. As the density normal to the channel (along the *y*-axis) varies insignificantly, we averaged the levels and number of particles along the *y*-axis for smoother representation of the results. The standard deviation along the channel was calculated as

(13) $$\sigma_{I}=\sqrt{\frac{1}{N_{y}}\sum_{J=1}^{N_{y}}{\left({\bar{PPC}}_{I,J}^{\left(TAS\right)}-{\bar{PPC}}^{\left(TAS\right)}\right)}^{2}},\;\;\;\;\;\;\;\;\;\;\;\mathrm{for}\;I=1,2,\ldots ,N_{x},$$

where ${\bar{PPC}}_{I,J}^{\left(TAS\right)}=\frac{PPC_{I,J}^{\left(basic\right)}}{level_{I,J}\times level_{I,J}}$ is the mean number of PPC in one TAS cell in a basic cell *I*,*J*, which is the number of particles in TAS sub-cells where the collision procedure was executed; ${\bar{PPC}}^{\left(TAS\right)}=\frac{1}{N_{x}N_{y}}\sum_{I=1}^{N_{x}}\sum_{J=1}^{N_{y}}\frac{PPC_{I,J}^{\left(basic\right)}}{level_{I,J}\times level_{I,J}}$ is the mean number of PPC in the computational domain per TAS cell; $N_{x}$ and $N_{y}$ are the numbers of basic mesh cells along the *x*- and *y*-axis, respectively; $PPC_{I,J}^{\left(basic\right)}$ is the number of particles in the *I*,*J* cell of a basic mesh; $level_{I,J}\times level_{I,J}$ is the number of cells of the TAS mesh in a basic cell *I*,*J*; and $level_{I,J}$ is the level of basic cell *I*,*J*. When PPC is equal to 2, four TAS levels exist in the basic mesh. When PPC is in the range of 3 to 10, three levels exist in the basic mesh. The solution is correct for PPC = 2, 3, 5, and 10 (see Figure 8). When PPC is equal to 20, the number of particles in a basic mesh is not sufficient and two TAS levels exist in a basic mesh. The results show that two TAS levels for the considered case are not sufficient and the velocity profiles deviate from the correct solution. When PPC is equal to 40, the TAS level in the basic mesh is one and the case corresponds to the solution without TAS. The velocity profile without TAS deviates from the correct one (see Figure 8). According to the results, correct solutions were obtained with TAS with at least three TAS levels in the computational domain. The standard deviation of PPC ($\sigma_{I}$) in the computational domain for PPC = 10 is up to 22, while for PPC = 20, it is around 35. The collision scheme obtains the correct results when the average number of PPC in the computational domain is within a given range to keep the number of PPC within a given range, one has to use appropriate TAS leveling or another type of adaptive mesh.

#### 3.1.4. The Computational Times of SBT and SBT-CDF Collision Schemes

The computational times (Central Processing Unit (CPU) time) of SBT and SBT-CDF collisional schemes are important and here, they are presented briefly. SBT-CDF involves more calculations than SBT. Nevertheless, the measured CPU times are the same or within a difference of up to 1.5 times. When we used TAS with PPC = 2 to calculate the pressure-driven flow, SBT-CDF needed the same computational time as SBT. For the case without TAS, the number of PPC is significant, but SBT-CDF needs around 1.5 times more CPU time than SBT. For the gas mixing case, presented later in the paper, the CPU times of SBT and SBT-CDF are equal.

#### 3.1.5. Validation of SBT and SBT-CDF Collision Schemes

The results obtained by DSMC using the collision schemes SBT and SBT-CDF were compared to the available results in the literature:

- An analytical solution of viscous, compressible isothermal flow in a long microchannel [25];
- A continuum model [26] based on Navier–Stokes–Fourier equations with appropriate velocity slip and temperature jump boundary conditions and calculated using the finite volume method SIMPLE-TS [27];
- The NTC DSMC collision scheme proposed by Bird [1].

The analytical solution (AS) [25], given for the pressure *p^AS^* (14) and the horizontal component of velocity *u^AS^* (15), can be rewritten in a non-dimensional form according to the given scales as follows:

(14) $$p^{AS}(x)=p_{out}\left[-r+\sqrt{r^{2}+\left(1+2\;r\right)\frac{x}{L_{ch}}+\left(P^{2}+2\;r\;P\right)\left(1-\frac{x}{L_{ch}}\right)}\right],$$

(15) $$u^{AS}(x,y)=-\frac{1}{5\;Kn\;\sqrt{\pi }}\frac{dp^{AS}(x)}{dx}\left[1+4{\left(\frac{y-0.5}{H_{ch}}\right)}^{2}+\frac{4\;F\;Kn}{p^{AS}(x)}\right],$$

where $\frac{dp^{AS}(x)}{dx}=-\frac{p_{out}(P-1)(P+2r+1)}{2L_{ch}\sqrt{{\left(P+r\right)}^{2}-(P-1)(P+2r+1)\;x/L_{ch}}}$, r=6 F Kn, and F=1 is the slip coefficient.

**Figure 10:**
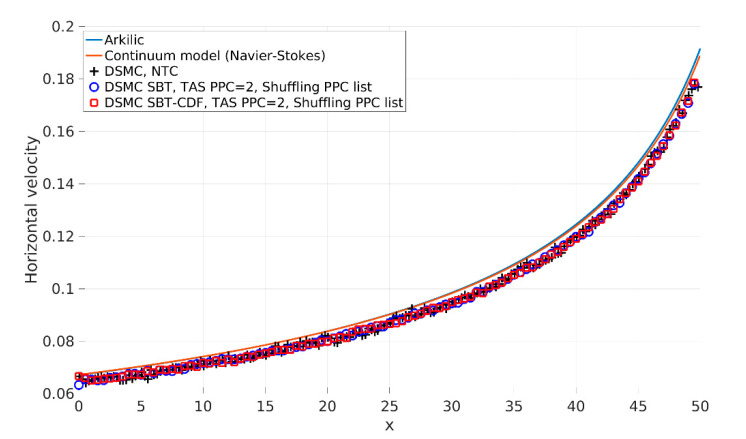
Horizontal component of the velocity along the centerline of the channel y = Hch/2. u-velocity profiles, from the analytical solution [25], continuum model (Navier–Stokes) calculated by the SIMPLE-TS method [27], Direct Simulation Monte Carlo (DSMC) NoTime Counter (NTC) collision scheme, DSMC SBT with TAS with PPC = 2, and DSMC SBT-CDF with TAS with PPC = 2.

**Figure 11:**
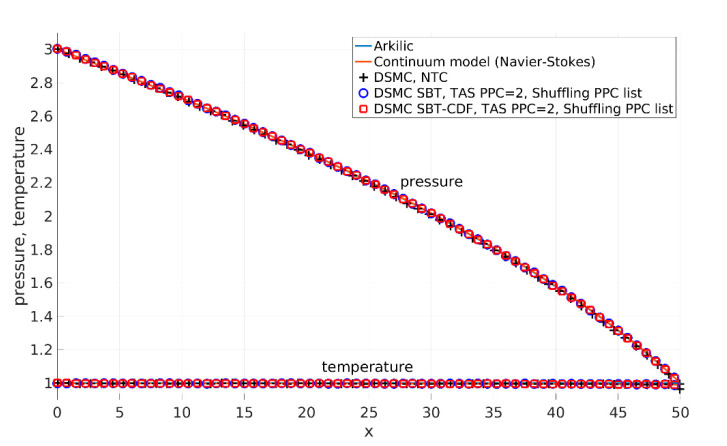
Pressure and temperature distribution along *x*-axis at the centerline of the channel y = Hch/2. Pressure and temperature profiles obtained from the analytical solution [25], continuum model (Navier–Stokes) calculated by the SIMPLE-TS method [27], DSMC NoTime Counter (NTC) collision scheme, DSMC SBT with TAS with PPC = 2, and DSMC SBT-CDF with TAS with PPC = 2.

We validated the presented collision schemes with an analytical solution, continuum model, and DSMC with a NTC collision scheme using a long enough channel. DSMC with SBT and SBT-CDF collision schemes used TAS with the condition of a minimal average number of PPC = 2. Figure 10 and Figure 11 present the results. Figure 10 shows the horizontal component of velocity along the centerline of the channel. The SBT and SBT-CDF collision scheme results are in perfect agreement with the others. For the considered Knudsen number, small differences between the DSMC and continuum model are expected (also pointed out by other authors, [1]). Figure 11 shows the pressure and temperature distribution along the centerline of the channel. The obtained results are in perfect agreement with the others.

### 3.2. Gas Mixing

**Figure 12:**
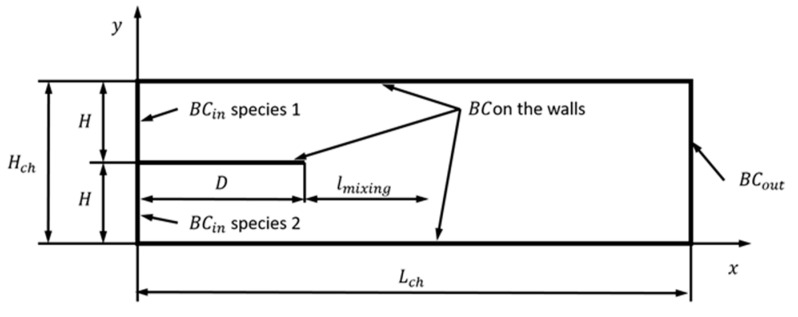
Geometry of a micro-mixer with length *L_ch_*, height *H_ch_*, and the inlet separator with length *D.*

The DSMC collisional schemes SBT and SBT-CDF were applied for calculating the mixing of two gases. The results were compared with those available in the literature. Figure 12 shows the geometry of the micro-mixer. The fluid flow was pressure–driven, as previously discussed, with an additional separation plate at the inlet. The channel length was $L_{ch}=8\;\mu m$, the length of the inlet gas separator was D=2 μm, and the half distance between channel walls was H=1 μm. The pressure at the inlet for species “1” and “2” was equal and fixed at $p_{in}^{\left(1\right)}=p_{in}^{\left(2\right)}=0.2\mathrm{atm}$ and was a reference pressure. Additionally, the temperature on the walls and at the inlet was equal to the reference temperature $T_{in}=300\;K$. The outlet pressure was equal to zero (expansion into the vacuum). Purely diffuse reflection was considered at the walls (the accommodation coefficient was equal to one). The intermolecular collision model was a hard sphere (HS) model. The reference Knudsen number was close to 0.3. Here, were mixed He and Xe. He entered from above (H≤y≤2H) and corresponds to area “1”. Xe entered from below (0≤y<H) and corresponds to area “2”.

**Figure 13:**
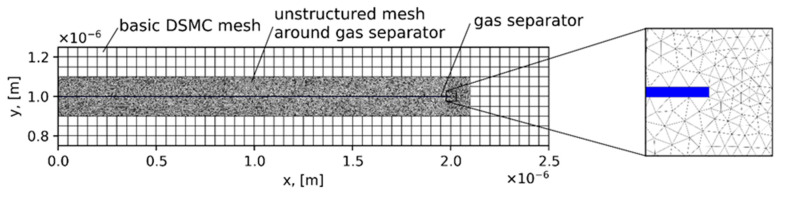
DSMC mesh near the inlet gas separator.

DSMC used an unstructured mesh near the inlet gas separator and a basic uniform mesh in the rest of the computational domain where TAS was applied (see Figure 13). A 2D adaptive unstructured mesh near the inlet plate was generated using the Delaunay algorithm by mesh generator Gmsh [28] with C++ API functions. This approach combines the fast indexing of particles in a uniform mesh and complex geometry with a minimal slow down. As we know, this approach was proposed for the first time in our previous paper [5]. DSMC used a basic uniform mesh with 160 × 40 cells and after the transition regime, 5.3 × 10^5^ particles of He and 4.0 × 10^5^ particles of Xe. In a basic mesh, we used TAS with an average number of PPC = 2. The presented results were time averaged based on 20,000 kinetic steps.

The results obtained using the SBT and SBT-CDF collisional schemes were compared to the results in [29]. The number of each species at the (*I*,*J*) cell is denoted as $PPC_{I,J}^{\left(basic)(a\right)}$, with *a* = 1, 2. It is compared relative to the number density difference $\xi_{I}^{a}$ defined in [29] as

(16) $$\xi_{I}^{a}=\frac{1}{N_{x}}\sum_{I=1}^{N_{x}}\frac{\left|PPC_{I,J}^{\left(basic)(a\right)}-{\bar{PPC}}_{I}^{\left(basic)(a\right)}\right|}{{\bar{PPC}}_{I}^{\left(basic)(a\right)}},$$

where ${\bar{PPC}}_{I}^{\left(basic)(a\right)}$ is the average number density of species *a* = 1, 2 of cells in column *I*, given by

(17) $${\bar{PPC}}_{I}^{\left(basic)(a\right)}=\frac{1}{N_{y}}\sum_{J=1}^{N_{y}}PPC_{I,J}^{\left(basic)(a\right)}.$$

**Figure 14:**
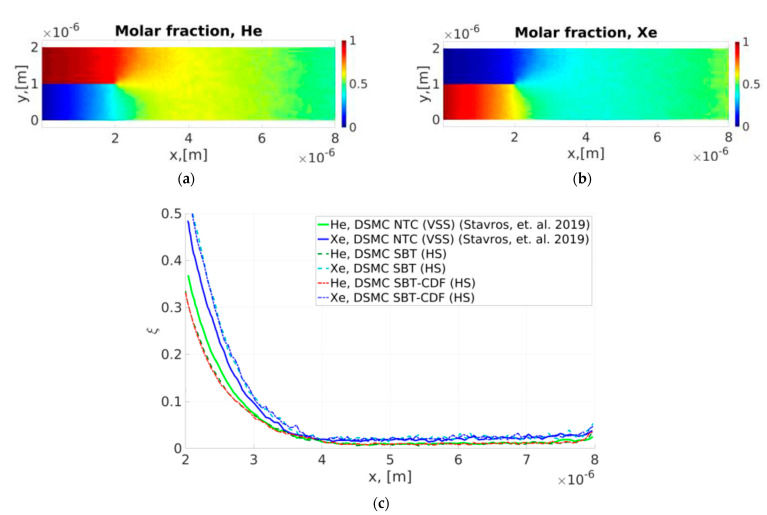
Gas mixing of He and Xe using the Hard Sphere (HS) model, (**a**) the molar fraction of He using the SBT collision scheme, (**b**) the molar fraction of Xe using the SBT collision scheme, and (**c**) a comparison of the relative density difference obtained using SBT and SBT-CSF collision schemes and the Hard Sphere (HS) model with the results available in [29] using the NTC collision scheme and Variable Soft Sphere (VSS) model.

Figure 14a,b show the molar fraction of He and Xe, respectively. The results were obtained by the SBT collision scheme. Figure 14c shows a comparison of the relative density difference of He and Xe. We compared the results obtained by SBT and SBT-CDF collision schemes using the Hard Sphere (HS) model with the results available in [29] using the NTC collision scheme and Variable Soft Sphere (VSS) model. SBT and SBT-CDF obtained the same results. The curves from 2 to 4 µm are slightly different for the HS and VSS models. Nevertheless, the mixing lengths and relative density differences after the mixing length of both models are very close.

## 4. Discussion

**Figure 15:**
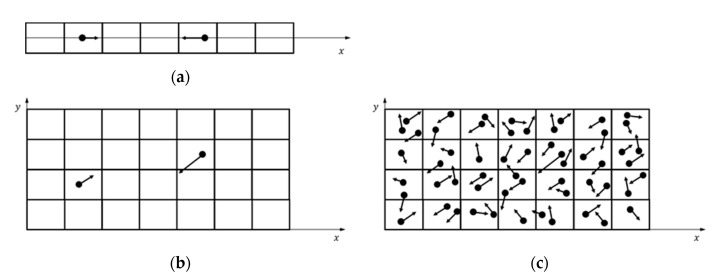
DSMC with a different number of PPC: (**a**) 1D case with a small number of particles per cell, (**b**) 2D case with an insufficient number of PPC, and (**c**) 2D case with a sufficient number of PPC.

We investigated the SBT collision scheme correctness for 2D cases. We selected the SBT scheme as a representative of BT-based schemes. Our aim was to obtain recommendations for the application of the SBT collision scheme for correct calculations of 2D flows. There is a fundamental difference between 1D and 2D (3D) cases. Successful 1D approaches and conclusions are not always applicable to 2D and 3D cases. To discuss the fundamental differences between 1D and 2D, we will use term boundaries, which means the boundaries of a considered region of the computational domain, such as a cell or a couple of cells. This can be extended to the boundary conditions of a problem if all cells of the computational domain are included. The important aspect here is the basic assumption of the DSMC method: Separation of the motion of the gas molecules into the ballistic and the collision parts. It is silently accepted that the particles have to be regularly included in both procedures. “Regularly included” means that particles move with velocity with a given distribution, representing a moving part, and have a given collision frequency that is obtained by a collision procedure. The gas properties correspond to the specified velocity distribution and collision frequency (mean free path). This is a rarefied gas simulation. When some of the particles miss procedures, the properties of the simulated gas are different from those specified and the obtained solution deviates from the correct one. We suppose that the time step is sufficiently small to ensure that none of the particles will fly over a cell without execution of the collision procedure (the CFL condition is satisfied). In 1D cases, boundaries are fully determined by one particle. Two neighboring particles with appropriate relative velocities cannot pass each other without participating in the collision procedure together (see Figure 15a). In other words, particles are included in moving and collision procedures proportionally. As a result, the simulated gas has a given velocity distribution and collision frequency. SBT exhibits an excellent accuracy for 1D flow, even for a small number of PPC (PPC = 0.5) [18]. In 2D cases, for the boundaries to be fully determined as in 1D cases, one has to use lines in boundaries, but we use particles, i.e., this is the fundamental difference in completeness of the definition of boundary conditions. Nevertheless, in satisfaction of the CFL condition, two neighboring particles with appropriate relative velocities can pass each other without inclusion in the collusion procedure (see Figure 15b). Such irregular passes happen when the number of PPC is not sufficient. This results in disproportions of moving and colliding procedures (the time step is included in the calculation of the probability of collision). As a result, we rarely execute the collision procedure, the collision frequency decreases, the mean free path increases, and the obtained solution deviates and corresponds to the solution with a greater mean free path. When the number of PPC is sufficient, the particles regularly participate in moving and collision procedures (see example with PPC = 2, Figure 15c). The comparison of profiles for 2D pressure-driven flow with PPC = 2 and PPC = 0.5 shows the described behavior (see Figure 7). The results of PPC = 0.5 deviated from the correct solution and correspond to the solution with lower collision frequencies (greater mean free path). Similar deviation of 2D cavity flow results exists in [7]. The exact minimal number of PPC is between 2 and 0.5 and could be found for a considered case after a series of time-consuming calculations and exact criteria for acceptable deviation. The results show that the average number of PPC equal to 2 guarantees the regular participation of particles in moving and collision procedures. We consider that PPC = 2 is sufficiently good and forms our soft recommendation for the minimal number of PPC.

We would like to mention that SBT is a relatively new scheme and has been gaining popularity in recent years. Questions regarding its applications and correctness of calculation of real problems are important. The problem of the minimal number of particles discussed here has never occurred for NTC scheme because it requires more than PPC = 5 for 1D flow to obtain the correct results [18]. That requirement is far from the PPC = 2 and PPC = 0.5 used here, where the considered disadvantage of the main assumption of DSMC occurs.

The smoothness of the number of PPC with TAS is important for SBT to obtain the correct solution. The results presented in Section 3.1.3 show that SBT obtained the correct results when TAS leveling varied from 4 to 3 in the computational domain, which corresponded to PPC ranging from 2 to 10. The number of particles in PPC lists used in the collision procedure has to be in a given range. One can use an adaptive mesh (TAS or another type) or uniform mesh when the number of PPC in computational domains varies in a given range.

The influence of the shuffling of the PPC list was studied. Particle rearrangement in the collision procedure was mentioned in [19], but without results showing the influence on the solution. We investigated the influence of shuffling of the PPC list on the algorithm that sorts particles in the PPC into groups, according to the cell that they came from. The underlying concept of Monte Carlo methods is to use randomness to solve problems. When the randomness is violated, the solution is not correct. In our case, the randomness is violated by the ordered or same PPC list in different time steps. The shuffling of the PPC lists is essential for correctness of the SBT collision scheme.

The same considerations are valid for other types of algorithms that obtain PPC lists in other ways. The algorithm used in Bird’s code obtains a significantly shuffled list of PPC because the particles are not grouped by its previous cell. Nevertheless, when in some regions in the computational domain, the particles in the list of PPC do not change for a sufficient number of time steps (where the local velocities are lower), SBT will generate fewer collisions. The results will deviate in a similar way as we have presented here. This makes the shuffling an important part of SBT and SBT-CDF schemes.

We followed the obtained recommendations and compared the obtained results produced by SBT and SBT-CDF collision schemes for binary gas mixtures of He and Xe. We used a small number of PPC (PPC = 2) and the HS model. The obtained mixing lengths and relative density differences were very close to those available in the literature that were obtained with the NTC collision scheme.

In recent years, the DSMC method has become widely used by non-professionals in this field because of the rapid development of MEMS. Bird’s book [1] is accepted as the main book about DSMC. A simple explanation of the collision scheme is missing. Interdisciplinary investigation results have demonstrated their importance. MEMS are complex devices and DSMC is the dominant method for modeling the gas in the transition regime. An accessible explanation of the collision scheme is useful to non-professionals interested in using DSMC and encourages interdisciplinary investigations. Considering this, we have presented the SBT-CDF collision scheme using the cumulative distribution function and following the survival equation.

The SBT-CDF collision scheme requires up to 1.5 times more computational time than SBT and obtains the same results. The reason for such a minor difference is that the collision scheme is part of the DSMC code and other calculations. Nevertheless, it is important to note that the CPU time of different codes depends on many factors. It is possible for the same DSMC scheme in different codes (for simplicity, let us consider serial codes) to demonstrate a difference in the CPU time of more than a couple of times on the same hardware. The efficiency of the DSMC code depends on many parameters, such as the calculated problem, the exact implementation of the algorithm, etc. Such a consideration is important, complex, and outside of the scope of this paper.

## 5. Conclusions

A simple representation of the DSMC collision scheme using the cumulative distribution function and following the survival equation has been presented in this paper. The correctness of SBT collision schemes for the modeling of 2D flows was investigated. The shuffling and correct number of PPC are essential to the correctness of SBT. Following the obtained recommendations, the results of the SBT collision scheme agree with NTC for 2D pressure-driven flow and binary gas mixtures of He and Xe. The proposed recommendations could be applied to 3D flows.
